# Editorial: Nutrition, diet and endocrinological health in female children and adolescents

**DOI:** 10.3389/fnut.2024.1459419

**Published:** 2024-07-15

**Authors:** Valeria Calcaterra, Stefano Stagi, Elvira Verduci, Gianvincenzo Zuccotti

**Affiliations:** ^1^Department of Internal Medicine and Therapeutics, University of Pavia, Pavia, Italy; ^2^Department of Pediatric, Buzzi Children's Hospital, Milan, Italy; ^3^Department of Health Sciences, University of Florence, Florence, Italy; ^4^Meyer Children's Hospital, Scientific Institute for Research, Hospitalization and Healthcare (IRCCS), Florence, Italy; ^5^Department of Health Sciences, University of Milan, Milan, Italy; ^6^Department of Biomedical and Clinical Science, University of Milan, Milan, Italy

**Keywords:** nutrition, children, adolescents, diet, endocrinology, female

Nutrients serve as precursors to key molecules involved in endocrine processes and thus exert significant control over physiological processes and biochemical pathways (1). Correct hormonal balances are fundamental for sustaining reproductive functions and fertility (1).

From birth until adolescence, the quantity, quality, and composition of available food has a fundamental influence on general health (2, 3). Maternal nutrition has a major impact on the growth and development of the fetus (4, 5) and fetal weight plays a significant role in programming the endocrine systems during critical developmental stages. Restricted prenatal growth has been associated with long-term alterations in the endocrine axes (3), which has an effect on adult health and metabolism and increases the risk of metabolic diseases like type 2 diabetes (T2DM) and obesity (6). Fetal macrosomia increases the risk of metabolic diseases in the future, including hyperinsulinism and T2DM (Calcaterra, Verduci et al.), both of which potentially affect endocrine and reproductive health (4) (Calcaterra, Verduci et al.).

Hormones, metabolism, and nutrition interact to support postnatal human growth, puberty, and overall health. Growth and timing of puberty are critical markers of nutritional status and overall health (7). Malnutrition in children with obesity can be caused by poor dietary choices, low-nutrient foods, sedentary lifestyles, educational challenges, and socioeconomic problems. Obesity in childhood increases the risk of chronic illnesses such T2DM, cardiovascular disease, and metabolic syndrome (8). During the transition from childhood to adolescence and sexual maturity, an incorrect nutritional status—whether in excess or deficiency, from a qualitative and/or quantitative perspective—can affect future reproductive health by influencing the timing and tempo of pubertal development and the hormonal control of ovarian function (9) (Calcaterra, Verduci et al.).

The aim of this Research Topic is to investigate the relationship between food and endocrine and reproductive health in females to promote good clinical practice and guide health workers in their support of patients with potentially harmful diets and eating patterns.

Grey et al. outline the factors influencing maternal, infant, and young child nutrition (MIYCN) in the Solomon Islands throughout the first 1,000 days of life. The authors describe a complex interplay of elements at both a macro level (globalization of the food system, shifting economies, and climate change) and a micro level (household and individual food choices). They conclude that improving MIYCN in the Solomon Islands would require multisectoral interventions that target macro- and micro-level variables (Grey et al.).

Hu et al. identify global trends in nutrition and gestational diabetes mellitus (GDM). They review the literature on GDM and nutrition from 2011 to 2021 and conclude that research in this area could have enormous potential. The authors stress the importance of cooperation among countries with disparate economies and suggest developing a common diagnosis criteria for GDM (Hu et al.).

Zheng et al. report that a high-fat diet plays a key role in the pathogenesis of colorectal cancer (CRC), and this effect on the gut can also occur in the offspring of mothers with a high-fat diet. Currently, CRC diagnoses are occurring at progressively younger ages worldwide, and the disease's prognostic outcome is not optimal. The impact of a maternal high-fat diet on the colorectal development of offspring has not been well-studied. Offspring may be more susceptible to colorectal carcinogenesis as a result of a number of alterations brought about by a mother's high-fat diet (Zheng et al.).

Calcaterra, Magenes et al. examine the hypothalamic-pituitary-thyroid axis in undernourished and overnourished individuals with eating disorders (ED), including obesity, chronic illnesses that afflict teenagers and young adults, with an incidence sharply rising in younger children. Obesity and ED are a continuum, and it is now clear that certain conditions, such as bulimia nervosa and binge eating disorder (BED), can precipitate one another and have similar consequences for nutritional, metabolic, and psychological wellbeing.

There is a significant correlation between controlling body weight and thyroid function. Underweight and overweight eating disorders (ED) patients often have thyroid abnormalities, which vary in severity and occur at different stages of the disease's course affecting the endocrine system and metabolic processes. In a few cases, thyroid dysfunction is the cause rather than the consequence of disease and can be reversed. Given the important role TH plays in mood regulation, metabolic control, cardiovascular risk associated with dysmetabolism, and energy expenditure, it is imperative to monitor thyroid homeostasis in ED patients to prevent severe organ dysfunction and psychological complications (Calcaterra, Magenes et al.).

Ramírez-Garza et al. evaluated oxidative stress and nutrition, lifestyle factors, emotion management, and metabolic syndrome (MetS) in 132 adolescents. They analyzed 8-isoprostane in spot urine samples in healthy, at-risk and with MetS adolescents. Urinary 8-isoprostane levels were significantly higher in the MetS group compared to the healthy group, positively correlating with body mass index, waist circumference, waist-to-height ratio, body fat percentage, blood lipid profile and glucose, emotional eating, and refined cereal intake (Ramírez-Garza et al.). Conversely, a negative association was found between 8-isoprostane and sleep duration and fish intake (Ramírez-Garza et al.).

Wang et al. conducted a systematic review and meta-analysis to evaluate whether skipping breakfast increases the risk of obesity or being overweight in children. Meta-analysis data shows that children and adolescents who skip breakfast are more likely to be overweight or obese, particularly with abdominal obesity, than those who eat breakfast.

Finally, Calcaterra, Verduci et al. stress the emerging role of nutrition as pivotal in shaping both endocrine status and reproductive capabilities (1–3). Female endocrine and reproductive health is significantly impacted by diet and hormonal balance. Nutritional and metabolic variables have a considerable impact on reproductive outcomes starting with fetal development (Calcaterra, Verduci et al.). A healthy diet full of vital nutrients is critical for preserving hormonal balance, avoiding reproductive problems, and preserving fertility. It is critical to acknowledge nutrition as a modifiable component in preventing unfavorable reproductive outcomes and promote the reproductive wellness of adolescent females adopting a holistic strategy that combines good eating and lifestyle choices.

## Author contributions

VC: Conceptualization, Writing – original draft, Writing – review & editing. SS: Conceptualization, Writing – original draft, Writing – review & editing. EV: Conceptualization, Writing – original draft, Writing – review & editing. GZ: Conceptualization, Writing – original draft, Writing – review & editing.
